# Understanding the links between resilience and type-2 diabetes self-management: a qualitative study in South Australia

**DOI:** 10.1186/s13690-017-0222-8

**Published:** 2017-09-21

**Authors:** A. L. Wilson, D. McNaughton, S. B. Meyer, P. R. Ward

**Affiliations:** 1 Flinders University, Bedford Park, Australia; 2 University of Waterloo, Waterloo, Canada; 3 Discipline of Public Health, Flinders University, Bedford Park, Australia

**Keywords:** Resilience, Social determinants of health, Self-management, Chronic disease management, Australia, Type-2 diabetes mellitus

## Abstract

**Background:**

Research conducted by Ward, Muller, Tsourtos, et al. (Soc Sci Med 72(7):1140–1148, 2011) has led to the development of the psycho-social interactive model of resilience, which reveals the interaction between individual resilience factors (i.e. coping, confidence and self esteem) and external resilience environments (i.e. employment, supportive family environments and health promoting policies) in facilitating the development of resilience. This present study explored the utility of this model of resilience for understanding how people self-manage type-2 diabetes.

**Methods:**

Data were collected via 14 semi-structured life-history interviews with women and men living with type-2 diabetes mellitus (T2DM). Participants varied according to socio-demographics (gender, age, education level, income) and were recruited based on their self-reported management (or lack thereof) of T2DM.

**Results:**

The inter-play of internal traits and external resources with additive and subtractive resilience strategies were consistent with the psycho-social interactive model of resilience. Self-management was influenced by life history. Differences in self-management and material disadvantage were also identified. Alongside increased disadvantage are higher levels of external barriers to self-management practices.

**Conclusions:**

This paper supports the concepts of additive and subtractive resilience strategies for use with diabetes populations; providing health professionals and policy makers with an increased understanding of how to recognize and foster patient resilience for the improvement of self-care, disease management and ultimately health outcomes.

## Background

Australian data reports that ~1 million people (4.4% of the population) had been diagnosed with Type-2 diabetes mellitus (T2DM) in 2014–15; an increase from 840,000 (3.8%) in 2011–12 [1]. If left untreated or poorly controlled, T2DM can lead to reduced quality of life, renal disease, amputation, blindness or death [2, 3]. In addition to the physical health consequences, T2DM is associated with $6 billion of healthcare costs, including carer and Commonwealth Government subsidies [4, 5]. There are many risk factors for T2DM inclusive of genetics, aging and social and environmental factors, the interplay of which is complex and still not well understood [6]. Several Australian studies identify barriers to diabetes self-management including knowledge, employment, older age, stigma, lack of resources and depressive symptoms [7–9]. T2DM is most commonly an adult onset disease, although in some settings, children and adolescents are presenting with the condition [6]. It is experienced by people who may be living alone, isolated or lacking social support [10]. In an Adelaide based study several participants noted that they were managing diabetes “entirely on their own with no support from family or friends” [11]. T2DM is also linked to social determinants of health including but not limited to socio-economic status, education and food security, as well as ethnicity, nutrition and aging [6, 12, 13]. In two studies, individuals with low incomes were found to be less likely to state that difficulties self-managing their diabetes were due to personal choices, and more likely to reflect extrinsic factors such as lack of accessibility to health services and affordability of healthy food [14, 15].

T2DM is primarily treated through medication and self-management via modification to diet, increased physical activity, or in some cases weight loss [16]. Self-management is defined as “an active, responsible process of care, in which the patient works to maintain his or her health in close collaboration with the health care personnel” [17]. In relation to T2DM, self-management is also used to describe how patients manage their condition in the form of exercise, diet and blood-glucose monitoring [18]. However, the self-management regime can be challenging for patients to undertake and practitioners to foster given that it requires significant shifts in day to day lives [11]. The shame and expectation of weight discrimination or reprimand can also result in patients evading health care or treatment [19, 20]. Within Australia, the potential role of obesity in some cases of T2DM is frequently referred to as an ‘epidemic’, that is bought on by an unhealthy lifestyle, which can further increase the stigma associated with T2DM [6]. For some, this results in individuals choosing to keep their condition a secret from their employers and colleagues for fear of being judged, dismissed or denied justified promotions; resulting in anxiety, omitting blood-glucose monitoring, delaying medications and avoiding medical appointments and social activities [21]. Several studies have suggested that stigmatizing particular conditions and practices can result in depression, which is in turn, strongly associated with reduced or poor self-management of T2DM [22–24].

Quantitative evidence suggests that resilience programs can have a positive influence on diabetes self-management [25–28]. A recent resilience-based diabetes self-management education program was shown to improve both psychological and physiological health in African-American adults with T2DM [23]. There is also substantial evidence for both self-management [13, 14, 29–31] and resilience [32–34] between socio-economic status (SES) groups, suggesting that socio-economic disadvantage can have a significant influence on self-management practices and resilience. Currently there are only two studies in Australia that have looked at resilience in individuals living with T2DM. Given the identified link between resilience and the self-management of T2DM, there are calls for greater research to understand how resilience is developed and sustained over a lifetime and specifically, how this manifests in T2DM management. Despite the existence of models of resilience, there is a lack of research focused on understanding and explaining the potential links between individual resilience factors, self-management practices and the external environments that support them, and in particular, with individuals diagnosed and living with T2DM [26, 35]. The present study therefore tests the utility of the existing psycho-social interactive model of resilience developed by Ward, Muller, Tsourtos, Hersh, Lawn, Winefield and Coveney [36] in understanding and explaining the self-management of T2DM. The conceptual model of resilience takes into account the internal attributes and external resources that influence resilience, the relationships between these factors and how they are developed (built or eroded) over the life course [36]. It also recognises the notion of biographical disruption and reinvention, in which a major life event and therefore causes a shift in identity. Self-management strategies were chosen as a focus for the paper as they can have a significant influence on health outcomes and are often malleable within the participant’s life [37, 38].

Qualitative research is a well-established platform for exploring and developing greater understanding of the underlying reasons, perceptions, meanings and motivations behind what people say and do [33, 39, 40]. Herein we analyze interview data from 14 individuals to explore the usefulness of Ward et als. Model of resilience for understanding how people self-manage diabetes, and provide insight into how resilience is developed throughout the life course to perhaps permit the development of resilience-development programs. The resilience strategies described by Ward, Muller, Tsourtos, et al. were classified as either additive, where individuals actively took on new activities or outlooks, or subtractive where they gave up something or removed themselves from situations or relationships [36]. Herein we used data documenting the life history of participants to identify and examine additive and subtractive resilience strategies used by participants in the self-management of type-2 diabetes, and how this may have influenced self-management practices. Additionally, we explore how resilience differs between those from high and low socio-economic groups. By investigating said relationships, this study will lead to an improved understanding of how to recognize and increase patient resilience in order to improve self-management and ultimately health outcomes. This paper concludes by discussing the utility of the resilience model with diabetes groups, suggesting areas which require further investigation and proposing potential changes to the framework.

### Conceptual framework

The concept of resilience has been identified as a key enabling factor required to improve health, whereby increasing community and individual resilience acts as a buffer against various forms of adversity [41]. Deveson describes resilience as “the ability to confront adversity and still find hope and meaning in life” differentiating it from other concepts such as coping or resistance [42]. According to Rutter, resilience is an active process whereby individuals are “successfully able to manipulate their environments to insulate them from the negative consequences of adverse events” [43]. Furthermore, resilience often results from the “exposure to adverse situations and risk” as opposed to the avoidance of risk [44]. As such, life history is thought to have an effect on resilience as it influences the development of skills and strategies that are drawn upon during times of adversity [44]. In addition, studies have shown that once individuals from vulnerable groups develop resilience, they are more likely to actively participate in health promoting activities [44].

**Fig. 1:**
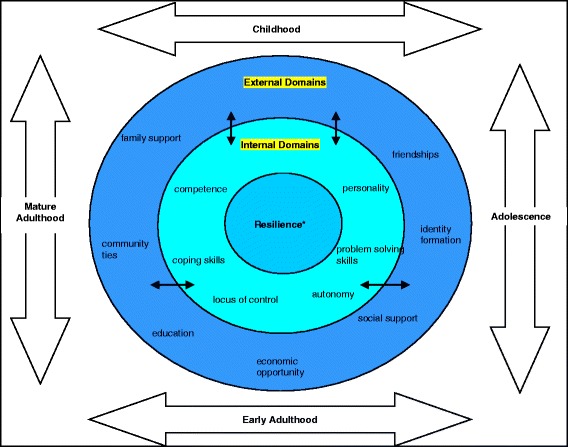
The psycho-social interactive model of resilience [36]. The small arrows in this model represent the two-way interaction between the internal and external domains. The large arrows represent the passage of time represented through life phases

The psycho-social interactive model of resilience developed by Ward, Muller, Tsourtos, et al. [36] shown in Fig. 1, is based on sociological research such as that by Bartley, Schoon, Mitchell, et al. [45] which identifies resilience as a set of conditions that allow individual adaptation to different forms of adversity at different points in the life-course, rather than property of the individual or something that is developed as a stable, personal characteristic. It also encompasses concepts from psychological resilience, which focuses on individual traits which make individuals more resilient, such as positive behavioral adjustment and the demonstration of behavioral competence [44].

When using the psycho-social interactive model of resilience to explore smoking, Tsourtos, Ward, Lawn, Winefield, Hersh and Coveney found that participants tended to “lack some of the internal and external resilience factors that the non-smokers reported” [46]. Further to this, Lawn, Hersh, Ward, Tsourtos, Muller, Winefield and Coveney [47] and Ward, Muller, Tsourtos, et al. [36] suggest that their results may be transferable to the general population and other at risk groups. Notably, Ward, Muller, Tsourtos, et al. [36] identified the role of ‘additive’ and ‘subtractive’ resilience strategies: the former included adopting new activities, roles or practices or a new positive outlook on life. For T2DM this would include exercise, changes in diets, taking part in community groups, diabetes education and peer support. Additive resilience strategies involve an inter-weaving of internal traits and external resources, with one “feeding off the other” [36]. Subtractive resilience strategies on the other hand, include moving away from those activities or practices regarded as reinforcing problematic or maladaptive behaviors. When applied to T2DM, these might include ceasing relationships with family, health staff and friends as well as poor dietary control, irregular monitoring, weight control, lack of regular exercise. The researchers also note that additive and subtractive strategies are often intertwined, as taking on one activity often means leaving behind another [36].

The notions of biographical disruption and reinvention were also explored in this study [36]. The diagnosis of T2DM is regarded as a major life event and therefore causes a shift in identity where by individuals are now ‘suffering from’ or ‘living with’ diabetes, where they were once considered free from diabetes or ‘healthy’ [36, 48]. This causes a ‘disruption’ and often-subsequent ‘reinvention’ where participants take up additive and subtractive resilience strategies to better manage their changing lives. This concept, its effects on self-management practices and differences in resilience between individuals will be discussed later in the paper. Finally, contemporary research on resilience focuses on an assets rather than deficits model, including factors leading to wellbeing and happiness [49, 50]. This approach attempts to understand the underlying social and psychological processes and practices, by which, resilience and ultimately improved self-management and well-being may be achieved.

## Methods

The present study involved 14 semi-structured life-history interviews with women and men living with T2DM. Qualitative research is a well-established methodological approach that is useful for understanding the experiences of individuals and how they bring meaning to situations, in this case, how they self-manage their diabetes [33, 39, 40]. The psycho-social model of resilience regards resilience as a trait that can be built up or diminished over time. As such, it was important to look at resilience within each participant’s life histories.

The sampling strategy was purposive and was developed to include individuals living with T2DM with varied abilities of self-management, as self-described. This sampling strategy allowed us to identify differences in self-management that may relate the individual resilience developed over the life-course (e.g. are those who identify as being able to manage more resilient?). Participants had varied socio-demographic characteristics including age, sex, highest education obtained, postcode and income to allow for stratification. Participants were recruited through two strategies; the first involved a mail out of 200 information packages to patients from a general practice in Adelaide. The second involved placing a flyer in Flinders In Touch, an electronic newsletter that is distributed to Flinders University Employees that ran for 6 weeks.

The interviews were conducted in 2013 and were structured based on Ward’s theoretical framework of resilience [36, 47]. The interviews were divided into life stages of early childhood, teenage years, early adulthood and mature adulthood. These life stages were seen to be distinct periods of biographical change where factors which inhibit, facilitate or diminish resilience could be identified, examined and understood [36]. Interviews were conducted face-to-face, by a single researcher (Sanderman) over a period of 6 months and took place in either the participants’ home or Flinders University. Questions focused on the participant’s life, relationships, self-management, goals, eating habits and key points in their lives such as developing independence and their diagnosis. Open-ended interviews allowed respondents to take the discussion in new directions, suggesting new meanings and reasons [18]. The oral-history format allowed for explorations and discussions of relevant experiences and perceptions of life history, biography and diabetes self-management [51]. It also allowed the participants to reflect on their past experiences throughout their lives, in order to allow them and the interviewer to interpret the factors influencing their motivations, behaviors and ultimately how self-management practices can be improved [52]. An excerpt of the interview questions is shown below:

Phase 1: Childhood (under 12 years).

Starting Question: Remembering your childhood:

- Can you tell me a bit about what it was like growing up when you were a young child?
  Probing Questions:

- How would you describe your childhood?
- Did you feel safe as a young child?
- Did you have a lot of people around you as you were growing up?

Diabetes related questions:

- As a child, what was a typical family meal?
- As a child, did people you live with have diabetes or any other chronic conditions that needed to be managed by adjusting lifestyle?
- What did you think about how they took care of themselves?

Interviews continued until data saturation was reached, and no new themes were occurring. Data saturation was reached after completing the fourteenth interview.

A thematic analysis of the data was undertaken, as it allowed the researchers to pin point, record and explore patterns within the data which was then used to describe the relationship between resilience and self-management [36, 51]. This approach increases the trustworthiness of the data as it moves beyond organizing and describing data to allow for an interpretation, providing a flexible and practical research tool to produce a comprehensive, detailed and complex account of data [53, 54]. A deductive approach was taken in the analysis in order to test the utility of the framework with diabetes populations [53].

The analysis was conducted lead by a single researcher (Wilson) using Nvivo 10 software and comprised three stages of coding; open, axial and selective coding. Open coding, in which themes (nodes) are established from the verbatim transcripts allowed the researchers to create a basic framework on which to base the analysis and address the aims of the study [54]. An example of open coding is provided in Table 1 below. This stage also involved reading over the verbatim interview transcripts and listening to the audio to become familiar with the data and potential themes. The established framework was then combined with a second framework, which was constructed using existing knowledge around self-management, resilience and the psycho-social interactive model of resilience.

**Table 1:** Examples of conceptual categories from open coding

Example of unit meaning	Conceptual theme	#
“It’s easy to change your habits if you want to.”	Control	139
“I didn’t understand a lot about type II diabetes, I figured that was just one of those things that happened.”	Responsibility	94
“When I split with my ex I said thank you for the diabetes, I thanked her for it. Because I know stress has a hell a lot to do with it”	Ownership	52

**Fig. 2:**
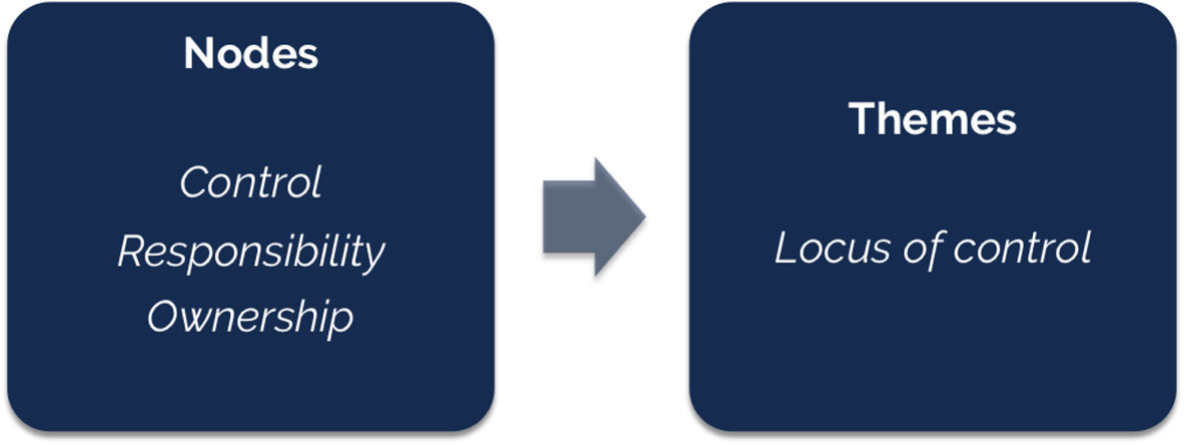
Example of axial coding, with theme ‘locus of control’ established from theoretical framework

The next stage, axial coding consisted of establishing relationships between categories and their relative sub-categories using existing literature and the psycho-social interactive model of resilience, to further determine relationships in the data [53]. An example of axial coding is provided in Fig. 2 below. In this phase of coding, the researcher linked the categories established in the open coding phase (internal/external factors, additive/subtractive strategies) to the sub-categories that contribute to it (education, community ties, social support, coping skills, locus of control autonomy etc.) [55]. This helped to establish a relationship between the variables and show how resilience and self-management are inter-related. A constant comparative method was used, in which the data collected was compared to previous knowledge around resilience, T2DM and self-management with codes added to the framework where necessary and as new themes emerged.

The final stage, selective coding, unified all existing categories around the ‘core’ category of resilience and self management, which represents the central phenomenon of the study [56]. Table 2 shows how the category 'locus of control' relates to resilience through both additive and subtractive, and internal and external concepts. In this phase, the researcher coded for resilience and self-management in the data. This is where the relationship between resilience and self-management of T2DM was developed. All authors took part in ongoing discussions around coding and emerging themes to reduce any possible bias within the analysis.

**Table 2:** Example of selective coding process

Category	Example	Concept	Global theme
Locus of control	Improve mental health	*Additive*	*Resilience*
	Removing negative friendships	*Subtractive*	
	Autonomy	*Internal*	
	Success of peers	*External*	
	Increase physical activity	*Additive*	*Self-management*
	Eating out less	*Subtractive*	
	Goal setting	*Internal*	
	Access to gym	*External*	

Participants are identified in the results using several socio-demographic factors including age, sex, SEIFA (Socio-economic Index for Areas) decile, self-diagnosed current self-management status (optimal or suboptimal) and life stage. Where information was missing for the participant, the field was left blank or replaced with n.d. (no data). The SEIFA index of advantage/disadvantage is constructed using a number of different variables that indicate advantage (high income, degree qualification) and disadvantage (unemployment status, low income) [57]. The appropriate SEIFA decile was determined using the participant’s residential postcode provided at the time of interview, with the 2011 SEIFA. The decile index was used as it clearly depicts areas of disadvantage, with a lower score indicating that an area is relatively disadvantaged compared to an area with a higher score [58]. Individuals with lower a SEIFA decile were considered to be ‘less disadvantaged’ than those with a higher SEIFA decile.

## Results and discussion

Several key themes were established from the analysis including; self-management strategies; resilience in the life-history of participants and its influence on self-management practices and; how resilience and self-management differ between those from high and low socio-economic status (SES) groups based on their SEIFA decile. These are reviewed throughout the discussion in relation to Ward’s framework in the form of: additive and subtractive resilience strategies; biographical disruption and reinvention and material disadvantage.

### Additive and subtractive resilience strategies

Overall participants identified more additive resilience factors, actively taking on new activities or practices (e.g. exercising, accessing specialist services, making different often healthier food choices, regularly monitoring blood glucose levels) for managing their diabetes than subtractive factors (e.g. going out to dinner less, buying less sugary food). These strategies involved the inter-weaving of internal traits such as; coping, autonomy and locus of control with external resources such as social support, community ties and education [36].

For example, after engaging in education sessions, several participants felt they had an increased understanding of the effect T2DM can have if untreated and were more confident in self-management strategies (e.g. eating a diet low in sugar, reading food labels, balancing meal portions, preparing the meal and timing). One participant spoke of the advantages of attending workshops at Diabetes SA (a member based organization that delivers services to people with Diabetes and their families) and how the support provided helped him to become more confident, to a point where he was able to manage on his own:

*Diabetes SA was a great support; they run lots of good information sessions. I joined up there, bought my gear there. Dieticians, that kind of stuff. After a couple of years I figured I was a smart guy and I’d done all the educating I needed. [Male, 56, optimal, SEIFA 8* ^*th*^ *decile, mature adulthood]*

The quote above demonstrates an additive resilience strategy (attending workshops), which positively influenced internal traits such as self-efficacy, ultimately improving self-management behaviors to avoid negative health consequences. Self-efficacy is described as the extent to which the individual believes in their own ability to complete tasks or reach goals [11]. Krichbaum, Aarestad and Buethe believe that self-efficacy is the link between knowing what to do and actually doing it and is influenced by observing others accomplish the task as well as verbal persuasion [59, 60]. Self-efficacy is demonstrated by this participant who discusses how his friend was able to stop taking medication and how he was influenced to do the same:

*I had another friend, who managed to go off anti-hypertensives just through exercise. So I thought about that sort of thing, would I potentially be able to go off the tablets. [Male, n.d., optimal, SEIFA 7* ^*th*^ *decile, mature adulthood]*

Self-efficacy has also been correlated to locus of control and self-esteem [59]. This concept is important for people with diabetes, as the level of self-management depends largely on the individual’s perception of their ability to perform activities with an expected outcome [61]. By seeing their peers have positive results from self-managing their conditions, it is possible for the individual to also undertake similar additive resilience strategies. This finding also supports previous research which demonstrated that Adelaide residents living with diabetes valued group education sessions for the knowledge gained, care and support provided and the social contact [11].

Internal resilience traits such as setting goals and developing carefully thought out plans are commonly associated with implementing additive resilience strategies, with external resources such as general practitioners (GPs), partners, education sessions, diabetic nurses used to increase motivation and self-determination. In the quote below, one individual describes how using the incentive of an overseas hiking trip (internal traits of goal setting and motivation) paired with support at a local fitness centre (external resource) helped him increase his physical activity levels and cope with mental illness (additive resilience strategy) and in turn improve how he lived with diabetes:

*Once I started to get some mobility back and then set myself the goal of going on a hiking trip overseas – and setting those positive goals helped with the depression… I started off with a relatively gentle program, saw a trainer for a few visits, one on one, working on a program for hiking and haven’t looked back. [Male, 56, optimal self-management, SEIFA 8* ^*th*^ *Decile, mature adulthood]*

Research suggests that when combined with visits to health professionals, internal resilience traits such as planning and goal setting can be strengthened to increase additive and subtractive resilience strategies [59]. Educators can help individuals to set short-term goals as described in the quote above, to help establish a pattern of success with self-management strategies; and improve internal traits such as self-efficacy, confidence, internal locus of control and ultimately resilience [59, 62].

Although the additive resilience factors of planning and goal setting described above were seen as a necessary step to successfully manage T2DM by participants, they were not undertaken by everyone. Several described avoiding their GP or dietitian due to a bad experience or relationship, which often stemmed from differences in self-management techniques. Examples provided by the participants included when the GP wanted a patient to take medication to aid self-management, but the patient wants to use dietary control; the patient wanting to cease a particular medication due to perceived side effects (weight gain, blurry vision, cough) but the GP does not agree; or a dietician advising the patient to consume a diet with fewer kilojoules than the patient believes they need. As the quote below demonstrates, other barriers to visiting health professionals included shame and fear of being placed on insulin, which is often linked to weight gain [6].

*I’m supposed to go, I’m due for my 3-month test now, but I’m too scared to go. I’ve got to go… I don’t want my doctor to say, ‘oh, I think we might have to think about insulin’. I don’t want insulin and I know the only way to stop that is to exercise and you know…so I’ve got to go and have my blood test done. [Female, n.d., sub-optimal, SEIFA 9* ^*th*^ *decile, mature adulthood]*

When discussing exercise with her husband, the participant also spoke of not wanting to go as she feels she is holding him back:

*And I feel that he’s been doing it for so long now and he walks fast. And I feel like I’m bringing him back. But he says, ‘I’ll walk with you and then keep going and doing my bit’. But that doesn’t work for me. [Female, n.d., sub-optimal, SEIFA 9* ^*th*^ *decile, mature adulthood]*

The above quotes on avoidance of health care professionals and exercise speak to a fear of insulin and avoidance of or delay in going to doctor for tests, which may be due to a sense of failure to manage and control diabetes and thus potentially shame. These concerns have been shown to be a common feature in delaying or avoiding medical visits and testing in a number of studies, which stated that shame and feelings of failure are significant barriers to self-management including studies in the local area [11, 61, 63]. These quotes also depict the relationship between internal traits and external resources, as one is influenced by the other.

Learning to make new choices (i.e. changes in food and exercise, linking in with health staff and education sessions) and implementing them into everyday life was a significant additive strategy for those managing diabetes effectively. Some were able to make this transition easier than others due in part, to skills learned earlier in life, or in other areas such as work. By working as a pharmacist, one individual felt confident to adjust recipes to make them suitable for a diabetic diet:

*The things I learnt in pharmacy set you up for that, because it’s all about following recipes, measurement, exactness. So those types of things I sort of learnt in a parallel way. So it wasn’t a big transition. [Male, ND, optimal, SEIFA 9* ^*th*^ *decile, early adulthood]*

This supports the idea presented by Harvey and Delfabbro, that previous experience is known to have an effect on resilience, as it influences the development of skills and strategies that are drawn upon during times of adversity [44]. Similarly, it has been suggested that resilience can be a ‘storehouse’ of tools and strategies, built up and used in future situations [36]. In this instance the changes (often significant in terms of food preferences, tastes and eating habits) were made easier, as this participant had already developed the knowledge, appropriate problem solving skills and self-assurance necessary to make the change. Another participant described his experience of moving out of the family home at a young age and developing his independence in the Navy:

*I went into the merchant navy when I was 17 and a half and so from the moment you go in there, you are looking after yourself. [Male, 80, optimal, SEIFA 5* ^*th*^ *decile, teenage years]*

This experience helped to foster resilience early in life, as similar to the pharmacist he developed skills (confidence, autonomy, problem solving skills) that could be drawn upon to self-manage his diabetes later in life.

While subtractive resilience strategies were less common, the relationship between external factors and internal traits was identified in several interviews. Once diagnosed with T2DM the following participant stopped purchasing and consuming what he viewed as unhealthy foods, ‘cutting back’ on behaviors he deemed unhealthy, and improving self-management practices:

*I stopped straight away. I cut back on everything, I didn’t smoke. I quit smoking close to 30 years ago. So it wasn’t an effect of that. But I cut everything back, so I started to go on oats and all that… [Male, 59, optimal, SEIFA 5* ^*th*^ *decile, mature adulthood]*

With the support of his local GP this individual was able to access useful resources (information & recipes) that he then applied to his personal life, increasing his coping skills. This quote implies that he already had a high internal locus on control, was aware of the steps he could take to better self-manage T2DM and believed he could influence the negative consequences of the disease. Locus of control refers to the extent to which individuals believe they can control events affecting them and is conceptualized as either internal (the individual believes they can control their life) or external (their decisions and life are controlled by environmental factors which they cannot influence) [43]. It is an important concept for self-management practices as increasing internal locus of control can improve individual’s ability to confront adversity [61, 64]. The quote from the participant also demonstrates how internal and external factors, additive and subtractive strategies are intertwined and in the case of T2DM can be dependent on each other as removing one often involves taking on another (i.e. cutting back on purchasing unhealthy food (subtractive strategy) involves purchasing healthier food (additive strategy).

Participants identified support from friends, family and social groups as a significant external resource, which positively influenced additive resilience strategies and ultimately internal traits. Friendships at local sporting clubs or having a ‘significant other’ encouraging them to ‘come for a walk’ increased physical activity levels for several individuals, which in turn increased self-confidence and optimism. In the quote below, the participant is describing how she tries to stay active, in order to keep up the maintenance of her garden and go on trips with fellow members of a local club:

*So yeah, I keep moving…See even my gardening, when I’m out there I’m constantly walking up and down. I’m part of a Pogonia’s society and we have excursions once a month looking at people’s gardens, travelling somewhere and it’s really very interesting [Female, 78, optimal, SEIFA 3* ^*rd*^ *decile, mature adulthood]*

This is supported by findings from earlier research, in which social connectedness has been demonstrated to be an important factor in facilitating better health outcomes in the long-term, and also has positive effects on their motivation to self-manage the disease [11, 65].

### Biographical disruption and reinvention

The concept of biographical disruption is significant in this study, as it describes major life changes that occur in response to the onset and management of chronic illness [36, 48]. For most this change was met with anger, sadness, fear or confusion, as shown in the quotes below:

*I’ve got friends who are huge and they don’t worry about what they eat and they haven’t got [diabetes], you know? And I think why haven’t they got it and why have I got it? It’s not fair. [Female, n.d., sub-optimal, SEIFA 9* ^*th*^ *decile, mature adulthood]*

*Was I [worried after being first diagnosed?] – Worried perhaps isn’t the right word; frustrated would be a better word. [Male, 56, optimal, SEIFA 8* ^*th*^ *decile, mature adulthood]*

Others welcomed the diagnosis as a new life challenge that provided an explanation for a series of symptoms they had been experiencing as the following statement suggests:

*Yeah, I felt really comfortable about it actually. Because it wasn't like I'd never heard to it before. It wasn't life threatening…But I don’t remember feeling upset about it. In fact I felt quite positive about it in one sense. Because I thought well that’s a bit of a challenge. [Male, ND, optimal, University, SEIFA 9* ^*th*^ *decile, mature adulthood]*

Notably, the ‘challenge’ of self-managing diabetes was more readily accepted by those with higher levels of education and/or social support.

Ward, Muller, Tsourtos, et als. Study on smoking cessation and resilience demonstrated that those who were physically healthy emerged as “more positive about life and more resilient to stressful life events,” in part because physical illness has psychological consequences [36]. This was also reflected by several of the individuals interviewed in this study who self-reported as physically healthy and did not have any life threatening illnesses. These individuals seemed to ‘bounce back’ from adverse situations better than those who were self-described as unhealthy or suffering from poor health (emphysema, oral health, loss of sensation in feet etc.). Several participants also spoke of how they are in ‘the best shape of their lives’ since being diagnosed with T2DM. The idea that these individuals are ‘healthy’ yet have been diagnosed with T2DM may speak to Crawford’s ideology of healthism, “the preoccupation with personal health as a primary – often *the* primary – focus for the definition and achievement of well-being; a goal which is to be attained primarily through the modification of life styles” [66]. It is possible that these individuals have identified themselves as healthy, as they have modified their lifestyles to a point in which they are no longer experiencing physical symptoms for their diabetes and therefore perceived themselves as having achieved some degree of health.

The biographical disruption framework while useful, fails to take in to account the positive sense of wellbeing or positive health outcomes that could be brought about following diagnosis, such as increased physical activity and consuming a more nutritious diet as suggested in the quote above. This insight lends itself to the concept of biographical reinvention, in which the ‘disruption’ leads to a shift in identity and a ‘reinvented self’ [36]. Biographical reinvention was presented through both additive and subtractive resilience factors such as *“throwing [themselves] into education programs” [male, 56, optimal, SEIFA 8th decile, mature adulthood]*, linking into community groups, and cutting out unhealthy foods that no longer fit their new biography. By gaining access to external resources and support networks many individuals were then able to ‘reinvent’ themselves and better self-manage their diabetes, and to define themselves as healthy and living with diabetes.

Interestingly, participants that had previously overcome major life changes such as quitting smoking earlier in life, appeared to be more equipped to self-manage their diabetes despite having a low income or being generally unwell. Self-management strategies such as; accessing help, adjusting purchasing habits and social ties with likeminded individuals were established, carried over in several cases from previous experience in quitting smoking and applied to the new diabetes lifestyle. This supports the concept of the psycho-social model as well as the ideas of Harvey and Delfabbro which suggest that overcoming adversity earlier in life allowed these individual’s to develop the skills they needed to make changes to their lives when faced with a diagnosis of diabetes [36, 44]. It is possible that undergoing biographical reinvention earlier in life (ie. quitting smoking), made it easier to reinvent themselves again.

Negative experiences, such as seeing a parent die from T2DM related illnesses or having friends on dialysis were also motivating factors and featured in discussions describing the kinds of biographical reinvention that had occurred. These experiences also led to biographical reinforcement where in, individuals took action to positively reinforce their new identities to prevent any additional negative health outcomes associated with the disease [44]. The example below demonstrates how one person became more self-aware, due to having seen her mother lose her leg to diabetes:

*Then she had one too many falls and went to hospital and went in a nursing home and then had her leg off. She hasn’t been right since….Oh yeah, I made sure I never got like that. I only have to see a sore on me and I’m off down the doctor. [Female, 62, sub-optimal, SEIFA 7* ^*th*^ *decile, early-mature adulthood]*

Despite having sup-optimal self-management practices, this participant accesses external resources such as her doctor regularly when she feels unwell and described a positive and supportive relationship with her spouse. This indicated that the she feels she can control some of the negative outcomes of diabetes, such as infections and amputation of limbs by being vigilant and seeking treatment early. The quote also demonstrates self-efficacy and awareness, where the individual is confident to access help when required in order to remain healthy. Resilience for this participant is discussed in more detail later in the paper; whereby a disadvantaged childhood had a significant influence on the development of resilience, which in turn had implications for her self-management practices. Although the participant is not yet optimally managing her diabetes, she is now beginning to develop the skills required to do so and may self-manage better in the future.

### Material disadvantage

Differences in SEIFA, life history and resilience were explored for potential implications on self-management practices. There was a strong external locus of control for those living in areas of higher disadvantage (as determined by the SEIFA decile), where individuals were more likely to encounter external barriers to self-management practices than internal. These external factors were both social and physical in nature, and influenced the individuals’ internal psychological traits (such as their perceived ability to control situations) as seen in the quote below:

*I’m not going to ride in when it’s raining, I’m not going to ride in when it’s 45 degrees and in Adelaide it’s either raining or it’s 45 degrees so that precludes me a lot from that. Next, there’s nowhere to store the bike here securely so that’s a bit of a worry. I mean I’ve got a room down the end there which actually is a switch room for all the networks and I could park it in there. Then I’ve got all this equipment I have to bring, so laptops and all the rest of it, so it’s just a heavy weight to do that. [Male, 53, optimal, SEIFA 3* ^*rd*^ *decile, mature adulthood]*

The individual describes various reasons (external factors) why he is unable to ride to work, despite living close by and purchasing two bikes in an effort to lose weight. Comparably, the female participant below from a less disadvantaged area, describes the interplay of internal barriers (motivation) and lack of external resources (social support) that prevent her from optimally self-managing diabetes:

*It’s not the lack of will and wanting to do it, it’s the motivation and being on my own I think. [Female, 62, sub-optimal, SEIFA 7* ^*th*^ *decile, mature adulthood]*

This is supported by earlier research, where individuals with low incomes were more likely to reflect extrinsic factors when discussing difficulties self-managing their diabetes than internal [14, 15]. It is also reinforced by previous findings that individuals are managing their diabetes without support from friends or family, which can lead to difficulties in self-management [11]. This may speak to differences in resilience between the two participants, where by less materially disadvantaged individuals may be more equipped, both physically and mentally, to undertake self-management practices.

As mentioned earlier, studies have shown that once individuals from vulnerable groups develop resilience, they are more likely to actively participate in health promoting activities [44]. One participant spoke of extreme levels of physical and mental illness within her family, beginning with her father when she was a child. Coping with ongoing adverse circumstances appears to have had an effect on her self-management practices and resilience, as despite being constantly busy and helping manage others’ conditions she is able to comfortably control her T2DM through exercise, taking part in a social sport team and preparing healthy meals. This demonstrates a strong sense of self-worth and high levels of coping skills. When describing her past experiences with managing her family’s health issues, the participant stated *“but life was like that, there was always something else [to deal with” [F, 78, optimal, SEIFA 3rd decile, early-mature adulthood].* The participant’s attitude towards recurring illnesses suggests that she believes they are out of her control and she is therefore unable to control them, demonstrating a high external locus of control. Instead, she copes and accesses external resources where possible for support, which improves her problem solving skills, competence and it would seem her health.

Although useful, the theory of resilience and health promoting activities by Harvey and Delfabbro does not apply to all vulnerable groups [44]. As Ward, Muller, Tsourtos, et al. noted, “growing up in materially deprived neighborhoods may increase the need for resilience in the face of increased adversity, but the likely assets and capabilities of people in those neighborhoods to develop resilience may be reduced” [36]. This theme was evident in the interviews between a couple that have both been diagnosed with T2DM but have significantly different self-management practices. Growing up in an unstable, disadvantaged household may have influenced the ability of one partner to self-manage her diabetes. She lacked external supports in childhood and resilience was not fostered, resulting in reduced confidence and autonomy. Agency is now given over to her husband, who optimally manages his diabetes through diet and exercise causing the participant to ‘misbehave’ when he is not around:

*I think it’s because I’m so closely watched here, I’m like a kid, you know? And I just feel like, ‘oh, blow it, he’s not here, If I want to have this, I’m going to have it. Because he watches everything, everything you put in your mouth he says, ‘you shouldn’t be eating that you shouldn’t be eating that’. [Female, ND, sup-optimal, SEIFA 9* ^*th*^ *decile, mature adulthood]*

Her husband actively uses support systems including their GP, Diabetes SA and Internet resources to improve his knowledge about T2DM. These additive resilience strategies have increased his self-confidence, internal locus of control and ability to self-manage both himself and his wife:

*At one stage we said right, she was taking metformin, a low dose. And I don't know whether it was put to us or we'd done it through reading. We could control it by diet, so then we started reading all the stuff about diet. The glycemic index, I got to know the ins and out of the carbohydrates. Then got certain books that recommended you have this for breakfast. So we tried that, and for a little while she was able to control her diabetes without medication. Then there was a lapse and she was back on medication. [Male, ND, optimal, SEIFA 9* ^*th*^ *decile, mature adulthood]*

It is possible that the female participant has made self-efficacy judgments and decided that she is not capable of managing her T2DM alone. Research suggests that judgments of self-efficacy determine how much effort individuals will expend and how long they will spend persisting in the face of obstacles or adverse experiences [67]. Bandura also notes that “when beset with difficulties people who entertain serious doubts about their capabilities slacken their efforts or give up altogether, whereas those who have a strong sense of efficacy exert greater effort to master the challenges” [67]. The quotes above suggest that the husband is confident in his abilities to self-manage his T2DM and has a stronger sense of efficacy than his wife.

As both participants have access to the same resources, previous life experiences clearly play a role in the resilience of these individuals and how they are able to self-manage their diabetes. It is possible that the male participant’s supportive family background where health was a priority and he learned to cook from a young age, played a role in developing internal traits and skills. Conversely, the female participant was left to her own devices as a young child and was bereft of the social and familial support that may have influenced her ability to develop the internal traits necessary to cope with the life changes associated with T2DM.

### Key messages

It is important to consider the individuals life history when establishing appropriate care plan. Educators and health care professionals can help individuals to set short-term goals to help establish a pattern of success with self-management strategies; and improve internal traits such as self-efficacy, confidence, internal locus of control and ultimately resilience.

1. When combined with visits to health professionals, internal resilience traits such as planning and goal setting can be strengthened to increase additive and subtractive resilience strategies.
2. Participants that had previously overcome major life changes earlier in life, appeared to be more equipped to self-manage their diabetes despite having a low income or being generally unwell.
3. There was a strong external locus of control for those living in areas of higher disadvantage (as determined by the SEIFA decile), where individuals were more likely to encounter external barriers to self-management practices than internal. These external factors were both social and physical in nature, and influenced the individuals’ internal psychological traits (such as their perceived ability to control situations).
4. Once individuals from vulnerable groups develop resilience, they are more likely to actively participate in health promoting activities. However, the capabilities of the individual to develop resilience may be reduced.
5. When experiencing difficulties people who entertain serious doubts about their capabilities slacken their efforts or give up altogether, whereas those who have a strong sense of efficacy exert greater effort to master the challenges.

Further research is required to develop our model into a more practical tool for use in clinical practice. The current model and method would require the clinician to undertake an oral history and probe the relevant domains of resilience, which is not feasible within the current time constraints of medical practice. Nevertheless, developing a practical tool would help clinicians to identify patients’ level of resilience.

## Conclusion

This paper explores the utility of the psycho-social model of resilience developed by Ward, Muller, Tsourtos, et al. for those living with T2DM self-management [36]. The inter-play of internal traits and external resources with additive and subtractive resilience strategies are presented and found to be similar to those highlighted in a smoking cessation study by Ward, Muller, Tsourtos, et al. and those in the original conceptual framework [36]. Life history, biographical disruption and reinvention were shown to have an influence on resilience as suggested in the framework, and were also seen to influence self-management practices later in life. Differences in self-management and material disadvantage are also described and it is argued that with increased disadvantage there are higher levels of external barriers, compared to internal ones. These factors can significantly influence the self-management practices and can be drawn upon by practitioners when working with individuals.

Economic opportunity was the only component of the psycho-social model of resilience that was unable to be applied, as no participants spoke to these issues. There was little data to suggest the influence of wider community resources on resilience, as most participants did not seek help outside of recommended health providers or close family and friends. However, given the well-established ties to social stigma it is not surprising that participants did not engage or seek wider support. In the future, when applying Ward’s framework to diabetes, adjustments could be made to include more diabetes specific items such as media as an external environment and internal traits such as perceived risk.

While other studies have investigated barriers and facilitators to self-management, resilience and diabetes, this paper has drawn together each of the components to bring about an improved understanding of how they are interrelated. Due to the qualitative nature of the study, claims cannot be made to the representativeness of the results outside of the study participants. However, this paper supports the concepts of additive and subtractive resilience strategies for use with diabetes populations; which can provide health professionals and policy makers with an increased and more nuanced understanding of how to recognize and foster patient resilience, in order to improve self-management, confidence and ultimately health outcomes. Future research should investigate the use of the psych-social model of resilience with other at-risk groups, to further establish its potential uses. Additionally, quantitative studies may wish to investigate the ability of the model to detect the same concepts of resilience in a questionnaire format. This could allow for practitioners and those supporting individuals with diabetes in their care plans, to ensure treatment is tailored to their abilities and experiences.

## Abbreviations

GP: General practitioner
SEIFA: Socio-Economic Indexes for Areas
SES: Socio-economic status
T2DM: Type-2 diabetes mellitus
